# Antigenemia and oral lesions in paracoccidioidomycosis: is there a correlation?

**DOI:** 10.1590/1678-7765-2025-0566

**Published:** 2026-01-19

**Authors:** Tatiana Teixeira de Miranda, Eva Burger

**Affiliations:** 1 Universidade Federal de Alfenas Instituto de Ciências Biomédicas Departamento de Microbiologia e Imunologia Alfenas Minas Gerais Brasil Universidade Federal de Alfenas (UNIFAL-MG), Instituto de Ciências Biomédicas, Departamento de Microbiologia e Imunologia, Alfenas, Minas Gerais, Brasil.

**Keywords:** Antigenemia, Paracoccidioidomycosis, Paracoccidioides brasiliensis, P. brasiliensis, circulating antigen

## Abstract

**Background:**

Paracoccidioidomycosis is a systemic mycosis caused by the dimorphic fungus *Paracoccidioides brasiliensis* and related species, which are endemic to Latin America. Oral manifestations are highly relevant for diagnosis, representing the primary anatomical site biopsied for confirmation.

**Objective:**

To investigate whether the virulence of *P. brasiliensis* influences the levels of circulating antigen in susceptible (B10.A) and resistant (A/Sn) murine models at multiple post-infection time points. The presence of oral lesions and their correlation with antigenemia were also assessed.

**Methodology:**

One-week-old female B10.A and A/Sn mice were inoculated with the *P. brasiliensis* lineages Pb18 or Pb265, whereas controls received sterile saline. At four, eight, 12, and 16 post-inoculation weeks, sera were collected via sub-axillary plexus incision under anesthesia. Circulating antigen levels were quantified using competitive ELISA. Specific antibody titers were determined by indirect non-competitive ELISA and *P. brasiliensis antigens*. Intergroup comparisons were performed using two-way ANOVA, followed by multiple comparisons. Oral cavities were visually examined by three independent evaluators to find mulberry stomatitis. Prevalence was expressed as the percentage of affected animals.

**Results:**

Significant differences in the circulating antigen levels were detected between susceptible mice infected with Pb18 and Pb265 and between susceptible and resistant mice infected with Pb18 16 weeks after inoculation. The highest levels of antibodies were found when both B10.A and A/Sn mice were infected with Pb18. During infection, no mice showed mulberry-like lesions in the oral cavity.

**Conclusion:**

The virulence of the infecting *P. brasiliensis* strain, although not specifically linked to the gp43 antigen, plays a critical role in antigenemia. Despite absent oral lesions-an inherent limitation of this murine model-this study provides relevant insights into the relationship between fungal virulence and circulating antigen levels.

## Graphical abstract

**Figure:**
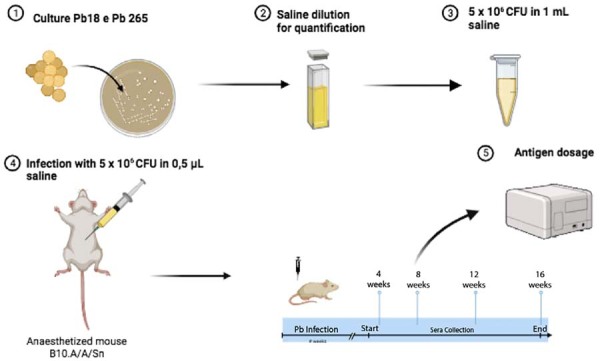

## Introduction

Paracoccidioidomycosis (PCM) is a severe systemic mycosis that is endemic to Latin America. It shows the highest incidences in Brazil, Argentina, Colombia, and Venezuela.^1^ The genus *Paracoccidioides* comprises several species that cause PCM. Advances in the 21st century have expanded our understanding of this genus, which now includes seven species: *Paracoccidioides brasiliensis sensu stricto*, *Paracoccidioides americana*, *Paracoccidioides restrepiensis*, *Paracoccidioides venezuelensis*, *Paracoccidioides lutzii*, *Paracoccidioides loboi*, and *Paracoccidioides cetii*. These species show different ecological and clinical characteristics. For instance, *P*. *brasiliensis sensu stricto*, *P*. *americana*, *P*. *restrepiensis*, *P*. *venezuelensis*, and *P*. *lutzii* affect the lungs and can progress to systemic granulomatous diseases.^2^

Haematogenic dissemination of the *P. brasiliensis* fungus from the lungs can originate secondary lesions in oral, rectal, and intestinal mucosae and the skin.^3^ Dentists play a crucial role in the early identification and management of PCM, particularly due to its oral manifestations. In fact, oral lesions are often the first visible signal of PCM, sometimes preceding pulmonary symptoms.^4,5^

Oral lesions progress slowly and tend to affect multiple areas, including the tongue, floor of the mouth, alveolar mucosa, gingiva, palate, lips, oropharynx, and buccal mucosa. These lesions appear as granular ulcers with hemorrhagic spots, a condition referred to as “mulberry stomatitis.” Periodontal involvement is also common, with the gingiva becoming red and swollen. Tissue destruction can lead to periodontal bone loss, exposed tooth roots, tooth mobility, and eventual tooth loss (resembling severe periodontitis). Lip swelling, known as macrocheilia, can also be observed.^6,7^

Although, PCM has characteristics that enable it to sometimes behave opportunistically, it occurs in immunocompetent and immunocompromised hosts. Certain factors, such as exposure to endemic areas, environmental changes, and possibly genetic predisposition, play a role in its manifestation.^1^ Interestingly, opportunistic PCM cases have been documented, such as in individuals with coexisting conditions that compromise immunity; although these belong to a broader context rather than the sole characteristics of the disease.^2^

The immune response appears to play a crucial role in the presentation of the clinical forms of the disease. Its severe manifestations, with the involvement of various organs and rapid evolution to death, are accompanied by the gradual loss of specific cellular immune responses and the high titer of specific antibodies. In comparison, its mild forms present fewer localized lesions, which increase healing and are parallel to maintained cellular immune responses and low levels of specific antibodies in patients and in mouse models.^8-12^

From an immunoregulatory standpoint, the prognosis of PCM reflects the cytokine profile predominantly expressed during infection. A good prognosis in PCM patients and resistant behavior in murine models correlates with the preferential secretion of Th1 and Th17/Th22 cytokines. Conversely, a bad prognosis in patients and susceptible behavior in the mouse model is associated with preferential Th2/Th9 cytokine production.^12^

Although the immune response is important in determining the progression of PCM, there is evidence of the involvement of *P. brasiliensis* antigens in the pathogenesis of this disease.^13,14^ Some studies have shown a circulating antigen in human PCM.^15,16^ The 43-kDa glycoprotein (gp43) is an antigen in PCM that is caused by *P. brasiliensis*.^16,17^However, antigenic variability exists between *Paracoccidioides* species and geographic regions.^18^ Such antigens are primarily involved in metabolic pathways, carbon metabolism, and secondary metabolite biosynthesis.^18^ The detection of these antigens (particularly gp43) in serum, cerebrospinal fluid, and bronchoalveolar lavage samples has shown high sensitivity and specificity for PCM diagnosis.^16^ The concentration of these components may also be correlated with the virulence of the fungal isolates.^19^

*P. brasiliensis* antigens may alter immune responses.^20,21^In the murine model of PCM, the alterations of immune response are associated with susceptibility. It is of interest to investigate whether circulating antigens are involved with susceptibility patterns.^22,23^ Thus, this investigation aimed to determine the *P. brasiliensis* circulating antigen in susceptible (B10.A) and resistant (A/Sn) mice that were inoculated with highly virulent (Pb18) and slightly virulent (Pb265) *P. brasiliensis* isolates and monitored at different time points after infection.

## Methodology

### Animal and ethical consideration

Week-old female mice of the highly susceptible B10.A strain and the highly resistant A/Sn strain were used in these experiments.^24^ The mice were given food and acidified-chlorinated water *ad libitum*. The review board for animal studies adopted by the Brazilian College of Animal Experimentation approved this study, which is in accordance with the Ethical Committee for Animal Research of the Biomedical Sciences Institute at the Federal University of Alfenas (UNIFAL-MG) under certificate number 0029/2023, Brazil.

### Fungi

Highly (Pb18) and slightly virulent (Pb265) *P. brasiliensis* isolates were used in this study.^25^ To prevent loss of virulence in the Pb18 isolate, sequential *in vitro* subculture for longer than three months was avoided. If a loss of virulence was found, then the fungus was submitted to passage in mice. Yeast-form samples from both isolates were cultivated in semi-solid Fava-Netto’s medium at 35 ºC. The fungi were used at their seventh day in culture. The yeast cells were washed three times in sterile saline and the fungal suspensions were adjusted to 10^7^ cells/mL after counting using a hemocytometer. The viability of the fungal cells, evaluated using Janus Green B stain, was always higher than 80%.

### Inoculation of mice

Animals were inoculated via an intraperitoneal route using 0.5 mL (5×10^6^ yeast cells/animal) of the relevant fungal isolate cell suspension, whilst controls received 0.5 mL of sterile saline solution. Each experimental group was composed of 6-10 mice for each strain (B10.A or A/Sn), infecting isolate (Pb18 or Pb265), and time of infection (4, 8, 12, and 16 weeks after inoculation), resulting in 16 experimental groups. The distribution of animals was as follows: G1 – 9, G2 – 10, G3 – 8, G4 – 8, G5 – 6, G6 – 8, G7 – 10, G8 – 10, G9 – 8, G10 – 8, G11 – 8, G12 – 7, G13 – 9, G14 – 9, G15 – 10, and G16 – 7 mice. Controls inoculated with saline solution were included in each experimental group.

### Collection of sera

The sera samples were collected from anesthetized mice at 4, 8, 12, and 16 weeks after inoculation by incision at the sub-axillar plexus. After coagulation, the sera sample were centrifuged at 1500 rpm and stored at −20 ºC until employed in the assays.

### Preparation of *P. brasiliensis* antigen (PbAg)

To obtain a standard antigen that could be used as a reference for subsequent analyses, yeast-form colonies from Pb265 and Pb18 isolates were cultivated in semi-solid Fava-Netto’s medium at 35 ºC. The yeast cells were ruptured at the seventh day of culturing. Yeast cells of *P. brasiliensis*, collected from the surface of the culture medium, were placed in a mortar and ground in an equal volume of finely powdered glass in three 10-minute stages, with 10-minute intervals between stages. All maceration steps were carried out on ice. The material was then resuspended in PBS (0.1 M, pH 7.2) with 10 mM of the protease inhibitor phenylmethylsulfonyl fluoride, homogenized, and left at 4 °C for 48 hours. Then, the suspension was centrifuged at 3000 rpm for 30 minutes at 4 °C, and the supernatant was collected, treated with Merthiolate (1:10,000), sterilized through a 0.22 µm membrane filter, aliquoted, and stored at −20 °C until use.

After the addition of 10 mM of the protease inhibitor phenylmethylsulfonyl fluoride (Sigma-Aldrich Corporation, St. Louis, Missouri, USA), the two antigens were mixed and subjected to SDS-PAGE immunoblotting analysis. Their carbohydrate and protein content was also determined.

Protein quantification of PbAg was performed according to the method in Lowry, et al.^26^ (1951), using bovine serum albumin (Sigma-Aldrich Corporation, St. Louis, Missouri, USA) as a standard at concentrations of 10, 25, 50, 100, 150, 200, and 250 µg/mL to construct the standard curve. To 0.4 mL of each sample, 0.2 mL of Folin–Ciocalteu reagent diluted 1:2 in distilled water were added. The mixtures were incubated for 30 minutes at room temperature and protected from light. Absorbance was then measured at 660 nm using a spectrophotometer. Samples containing distilled water were used as negative controls.

Carbohydrate quantification was performed according to the anthrone method^27^ using glucose (Sigma-Aldrich Corporation, St. Louis, Missouri, USA) as a standard at concentrations of 12.5, 25, 50, and 100 µg/mL. To 1 mL of each sample, 0.2 g of anthrone (Sigma-Aldrich Corporation, St. Louis, Missouri, USA) dissolved in 100 mL of 2.5% sulfuric acid were added. The mixture was boiled for 10 minutes and then cooled on ice for 5 minutes. Subsequently, absorbance was measured at 600 nm using a spectrophotometer. Distilled water was used as the reaction blank instead of the sample.

### Preparation of purified mice anti-*P. brasiliensis* IgG

To assess the reactivity of the Pb antigen, we prepared purified mice anti-*P. brasiliensis* IgG. Sera from 50 mice infected with Pb18 (confirmed by serology) were pooled. The IgG fraction of this pool was obtained by precipitation with 33% ammonium sulphate. The mixture was constantly shook for 30 minutes. Then, the precipitate was resuspended in an equal volume of phosphate buffer at pH 7.2 (PBS). The solution was dialyzed against PBS for 72 hours and subjected to ion exchange chromatography using DEAE Sephadex A-50 (Pharmacia, Uppsala, Sweden). The purified IgG fraction was evaluated by immunoblotting using the *P. brasiliensis* antigen above to assay its specificity.

### Competitive ELISA

The antigen levels in the sera samples were quantified by competitive ELISA, which was performed following the manufacturer’s instructions with some modifications. Briefly, a reference standard serum was prepared by adding PbAg to a normal mouse sera pool at a concentration of 50 µg/mL. Then, the reference standard was diluted using a buffer containing 0.01 M PBS (pH 7.2), 0.05% Tween 20, 5% defatted powdered milk, and 20 mM MgCl_2_. The resultant final antigen concentrations were 12.5, 3.1, 0.75, 0.2, 0.05, 0.013, and 0.0033 µg/mL. The reference standard was used to construct the standard curve for each plate separately.

Polystyrene plates were sensitized with 50 µl of PbAg diluted in 0.2 M sodium carbonate buffer (pH 9.6) containing 20 mM MgCl_2_ at a final total protein concentration of 1.0 µg/mL. The tested sera from infected and non-infected mice were diluted from 1:2 to 1:32 by serial two-fold dilution in buffer. Afterwards, each dilution from mouse sera and reference standard serum was incubated with an equal volume of mice anti-*P. brasiliensis* IgG that had been diluted 1:40 in diluent buffer.

Aliquots (50 µl) of each mixture were added in duplicates to previously sensitized wells and incubated for 2 hours at 37 ºC in a humidity chamber. The plates were washed thrice with PBS, and 50 µl of the anti-mouse IgG-peroxidase conjugate (Sigma-Aldrich Corporation, St. Louis, Missouri, USA) were added. The reaction was developed by adding 100 µl of a solution containing peroxide and *ortho*-phenylenediamine (Sigma-Aldrich Corporation, St. Louis, Missouri, USA), followed by the addition of 25 µL of 2N sulfuric acid (Merck KGaA, Darmstadt, Germany) to stop the reaction. Absorbance at 490 nm was measured using a microplate reader (Bio-Rad Laboratories Incorporation, California, USA).

### Non-competitive ELISA

Specific antibody titers of sera collected from infected mice were determined using an indirect non-competitive ELISA and the *P. brasiliensis* antigen. Polystyrene plates were sensitized with PbAg as described. Briefly, the mouse sera were first diluted to 1:40, and then by serial two-fold dilution in diluting buffer. Aliquots (50 µl) of each dilution were added in duplicates to previously sensitized wells. The plates were left for 2 h at 37 ^o^C in a humid chamber. After washing three times with 0.01 M PBS, pH 7.2, 0.1 mL of the anti-mouse total immunoglobulin peroxidase conjugate (Sigma) diluted 1:500 in diluting buffer were added, and the plates were incubated for 2 h in a humid chamber at 37^o^C. The plates were washed again, and the reaction was developed as described.

### Oral cavity examination

To assess mulberry stomatitis, the mice were gently restrained, and their oral cavities were inspected under adequate lighting conditions. Visual examination was systematically conducted, assessing the dorsal surface of the tongue, buccal mucosa, and the palate for characteristic lesions such as multiple small, raised nodules resembling a mulberry-like texture.

In total, three independent examiners who had been blinded to the experimental conditions visually inspected the oral cavity of each mouse. Each examiner recorded the presence or absence of lesion and their severity and distribution. Discrepancies in observations were solved by discussions, and consensus was reached before final data recording.

Findings from the three independent examiners were compiled and analyzed for inter-examiner agreement using Cohen’s kappa statistic. The prevalence of mulberry stomatitis was determined by the percentage of mice affected within the examined population.

### Statistical analysis

Statistical analyses were performed on GraphPad Prism, version 8. Differences in antigen and antibody mean concentrations between groups were tested using two-way ANOVA with multiple comparison tests. A statistically significant difference was considered if p<0.05. The same statistical tests were applied to analyze the percentages of animals found positive for circulating antigens under the same significance level.

## Results

### *P. brasiliensis* antigen and purified mice anti-Pb IgG analysis

**Figure 1:**
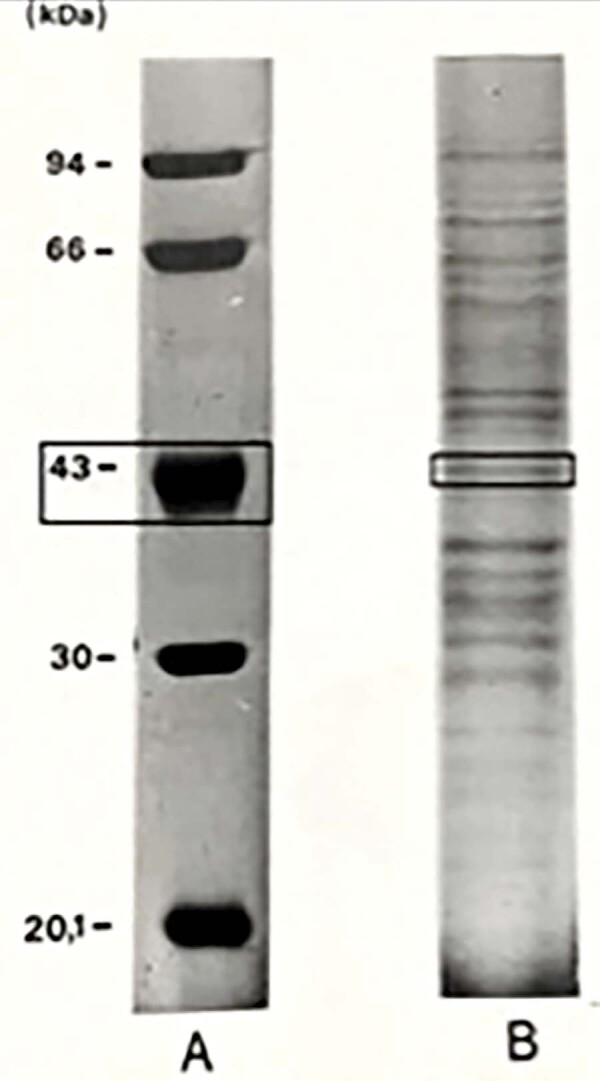
SDS-PAGE following silver staining, showing the 43kDa glycoprotein fraction of the antigen. A – Molecular weight marker; B – PbAg

**Figure 2:**
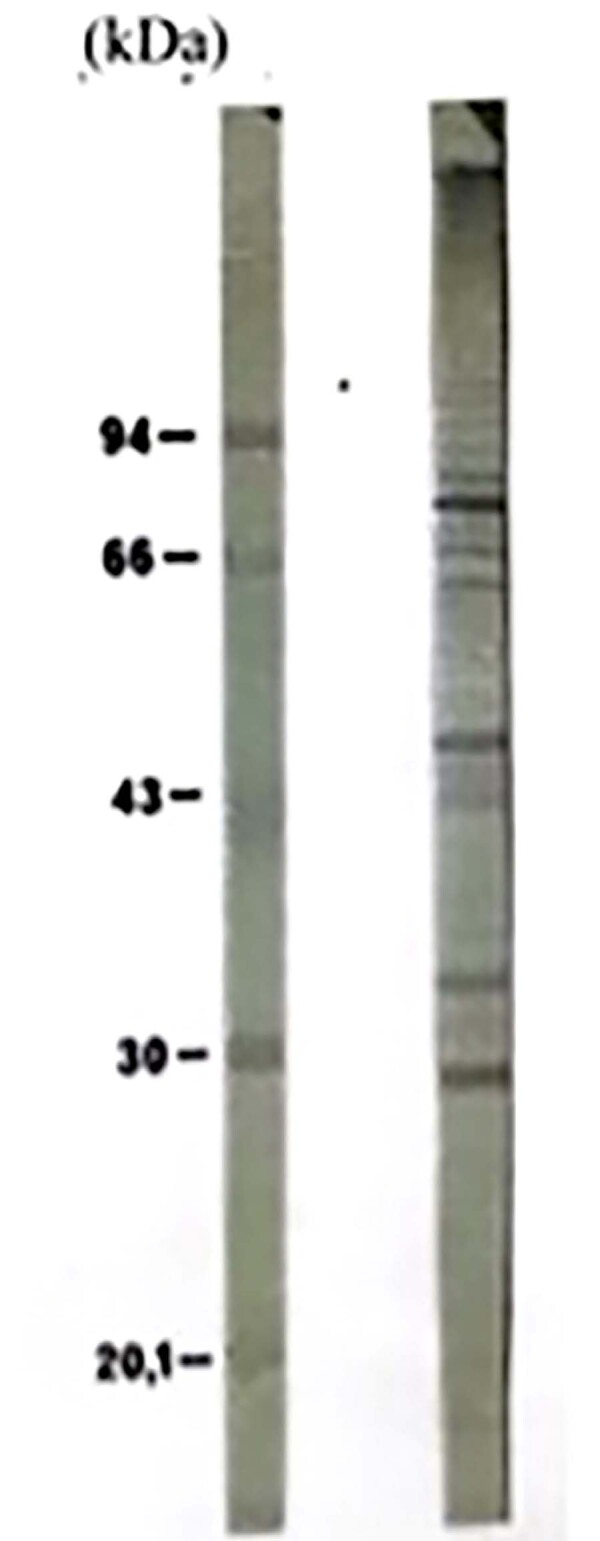
Protein fractions of PbAg recognized by mice anti-*P. brasiliensis* IgG using the immunoblotting technique. A - Molecular weight marker; B – Antigen reactivity.

Protein and sugar content of PbAg were 4.0 and 4.25 mg/mL, respectively. SDS-PAGE showed protein and/or glycoprotein fractions with different molecular weights in PbAg, including the immunodominant gp43 band of *P. brasiliensis* (Figure 1). The analysis by immunoblotting of PbAg and the purified mice anti-Pb IgG confirmed that this IgG found many PbAg fractions; the most evident of which were those of 74, 70, 66, 64, 50, 43, 35, 32, and 28 kDa (Figure 2).

### Percentage of mice confirmed with *P. brasiliensis* circulating antigen

**Figure 3:**
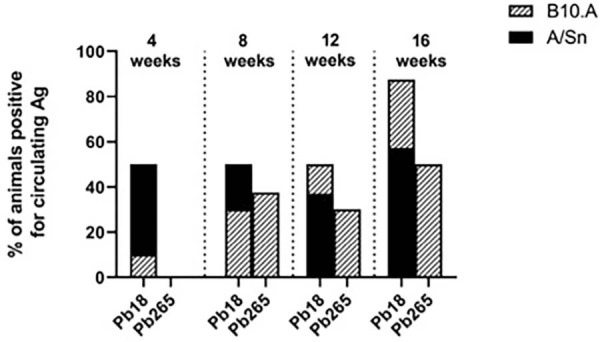
Percentage of mice with *P. brasiliensi*s circulating antigen.

The percentage of mice with confirmed presence of *P. brasiliensis* circulating antigen was analyzed after inoculation with either Pb18 or Pb265 isolates (Figure 3). The results show that when Pb18 was the infecting isolate, mice (both types) always showed a percentage of mice circulating antigen. This percentage increased with the duration of infection in the susceptible strain going from 10% in the 4^th^ week to 87.5% in the 16^th^ week, remaining constant and in lower levels in the susceptible strain than in the resistant one. On the other hand, when Pb265 was inoculated, no A/Sn mice was positive to the circulating antigen in the 4^th^ week, despite an increasing percentage of positive B10.A mice from the 8^th^week onward.

### *P. brasiliensis* circulating antigen levels in mice sera during infection

The standard curves that quantified the antigens in the mouse sera were obtained by different concentrations of PbAg. The sensitivity of the competitive ELISA to detect antigenemia in mouse sera infected with *P. brasiliensis* ranged from 0.051±0.043 to 14.4±3.64 µg/mL. The individual levels of circulating PbAg in almost all mice infected with Pb265 were generally lower than 0.1 µg/mL. However, some individual measurements reached slightly above this value, particularly around the 12th week post-infection. This contrasts with those infected with Pb18, which showed antigenemia ranging from about 0.2 to 0.6 µg/mL, including individual values that exceeded the group mean, particularly at later time points.

**Figure 4:**
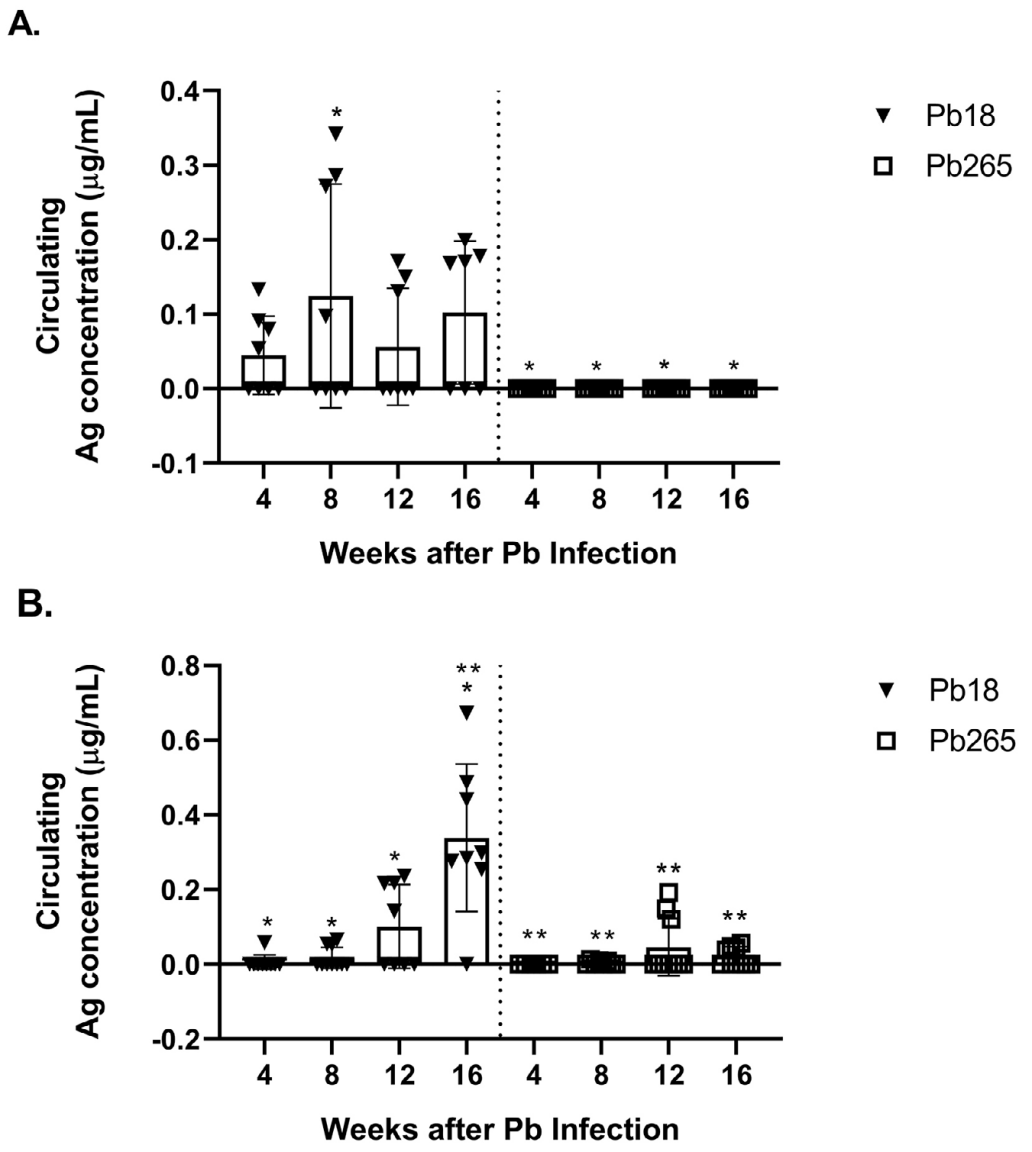
Mean antigen levels in A/Sn (A) and B10.A (B) mouse strains during infection with *P. brasiliensi*s isolates Pb18 and Pb265. (*) and (**) – p<0.05.

The mean antigen level in both mouse strains during the infections by the Pb18 and Pb265 isolates are shown in Figure 4A and 4B, respectively. A/Sn mice showed no positive results when infected with Pb265. When infected with Pb18, their geometric mean levels of circulating antigen remained consistently low and oscillated throughout the experiment. In contrast, B10.A mice infected with Pb265 showed low antigen levels, whereas infection with Pb18 resulted in initially low levels (fourth and eighth weeks) that progressively increased, reaching the highest values 16 weeks after inoculation. For both isolates, all data points (including outliers) were considered in the mean calculations to ensure result consistency and comparability.

Significant differences (p<0.05) in circulating antigen were obtained between B10.A mice infected with Pb18 and Pb265 and between B10.A and A/Sn mice inoculated with Pb18 at the 16^th^ week after infection.

Overall, both mouse strains showed higher levels of circulating *P. brasiliensis* antigen when inoculated with the highly virulent Pb18 isolate than with the less virulent Pb265 one. The levels of circulating antigen increased as the disease progressed in the susceptible strain, reaching peak values 16 weeks after infection with Pb18. Conversely, antigen levels in the resistant strain (A/Sn) remained low and oscillatory throughout the infection, remaining below the detection limit in mice infected with Pb265.

### Total specific anti-*P. brasiliensis* antibody titers in mice sera during infection

**Figure 5:**
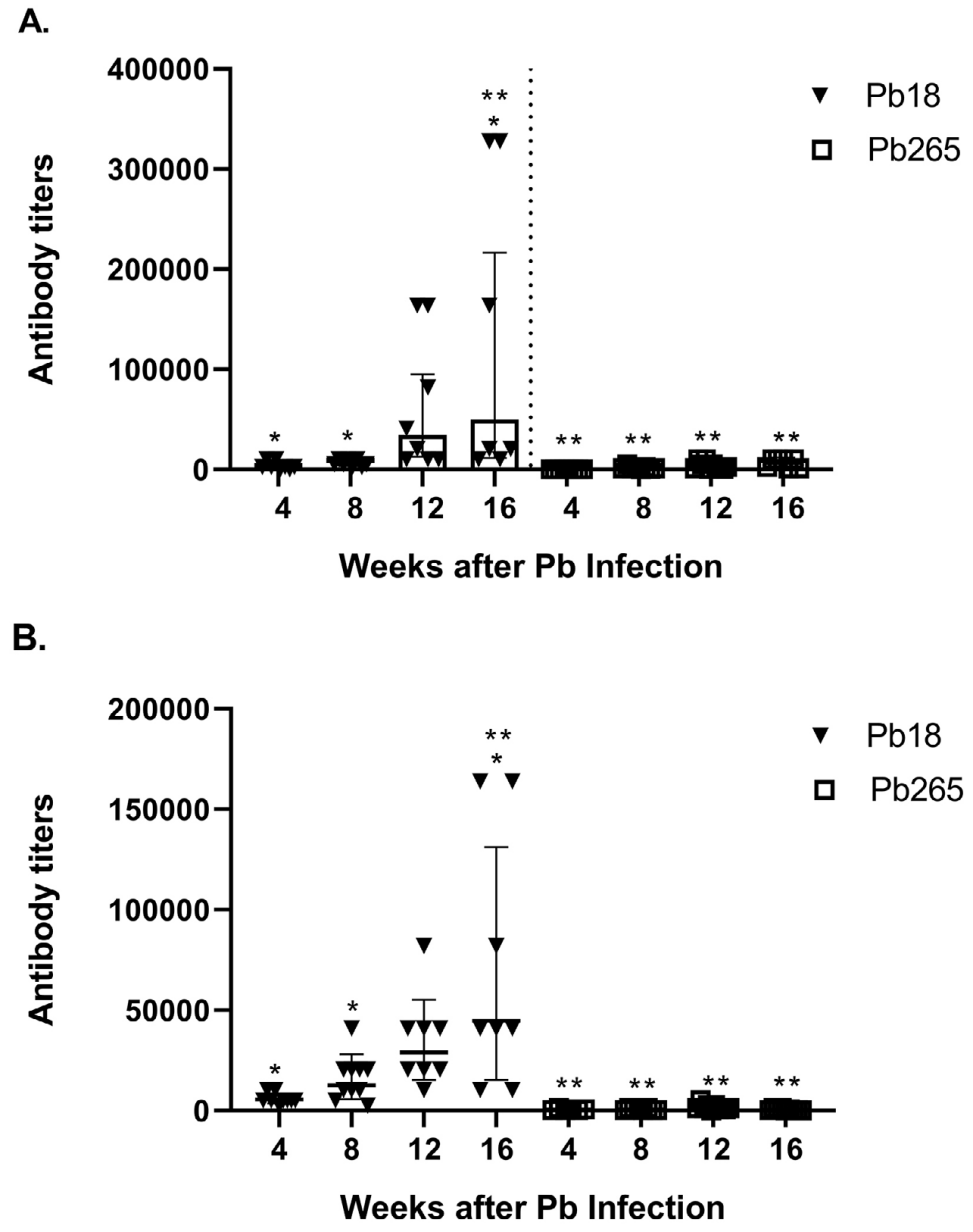
Total specific anti-*P. brasiliensi*s antibody titers in A/Sn (A) and B10.A (B) mouse sera during infection with Pb18 and Pb265 isolates. (*) and (**) – p<0,05.

Higher antibody titers were obtained in the B10.A and A/Sn mouse groups after inoculation with Pb18 than in the groups infected with the Pb265 isolate (Figs 5A and 5B). The antibody titers were low after the 4^th^ week and increased progressively up to the 16^th^ week after inoculation with Pb18 in both mouse strains. When the B10.A and A/Sn mice were infected with Pb265, the antibody titers were always low in both mouse strains in the course of the disease. Significant differences between B10.A and A/Sn mice were absent when the animals were infected with either isolate.

Significant differences were detected between the B10.A mouse group infected with Pb18 and the B10.A group infected with Pb265, across all time points. Significant differences were also observed between the A/Sn mouse group infected with Pb18 and the A/Sn group infected with Pb265 four, 12, and 16 weeks after inoculation.

### Detection of mulberry-like lesions in the oral cavity

No characteristic lesions were observed in the oral cavity during infection, regardless of the type of microbial isolate or animal strain at any studied infection times. Cohen’s Kappa coefficient was 0.95, indicating almost perfect agreement between the examiner’s opinions regarding the absence of mulberry-like oral lesions.

## Discussion

Our study shows that the fungal virulence and antigenemia in PCM are fundamentally connected to the pathogenicity of *P. brasiliensis* and hosts’ immune responses, but it was impossible to correlate antigenemia with oral lesions occurrence. In our experimental model using virulent (Pb18) and less virulent (Pb265) isolates of *P. brasiliensis*, mice infected with the more virulent Pb18 strain showed higher levels of circulating fungal antigens (antigenemia) than those infected with the less virulent Pb265 strain. This suggests that fungal virulence directly influences the degree of antigenemia.

Given these observations on fungal virulence and antigenemia, it is also important to address a specific methodological aspect of this study—its exclusive use of female mice. The choice of females was intentional and based on biological and methodological criteria. The animals were one-week old, a prepubescent stage at which circulating estrogen levels remain basal and exert none of the hormonal protection in adult females. Therefore, hormonal influence on infection establishment or progression is negligible at this age. Moreover, the fungal inoculum consisted exclusively of the yeast form of *P. brasiliensis* (Pb18 and Pb265 isolates), prior to the mycelium-to-yeast transition that is inhibited by female sex hormones. By using young female mice, we ensured a hormonally stable and reproducible model, minimizing biological variability and avoiding aggressive or stress-related behaviors commonly observed among males.

The results suggest that the circulating antigen levels have a correlation with the spread of the disease, and that this phenomenon is related to the virulence of the *P. brasiliensis* isolate used to infect the mice. Previous studies on the influence of the *P. brasiliensis* isolate virulence over infection of B10.A mice have shown that Pb265 isolate causes few granulomatous lesions in the parenchyma of the organs, with high lethal dose (LD_50_) and Pb18 isolate causes disseminated granulomatous lesions and low lethal dose (LD_50_).^28,29^ Considering that Pb18 isolate disseminates to many organs, these fungi may release metabolic products into hosts, which would enable the detection of high circulating antigen levels after Pb18 isolate infections. Borges, et al.^30^ (2023) used transcriptional and proteomic studies of yeasts in the pulmonary granulomas of PCM. The authors reported an increased expression of transcripts for CTLA-4, PD-1, and arginase-1, which provided evidence of immune regulatory mechanisms within the granulomatous lesions.

On the other hand, Pb265 produce low antigen levels in B10.A mice and none in A/Sn mouse sera. Also, Siqueira, et al.^31^ (2016) have shown distinct patterns of host-parasite interaction and pathology caused by Pb18 isolate.

Evidence from previous studies has indicated that patients with PCM had the highest frequency of positive antigenemia and that the highest antigen level occurs in patients with the acute form of the disease.^32,33^ These results agreed with the disseminated form of the disease in such patients. This aspect resembles that in the susceptible mouse strain at late phases of infection with Pb18. Whilst the B10.A and A/Sn mice model fails to mimic what happens with patients with the acute form of PCM, it reproduces the severe and mild forms of chronic disease, respectively. Quantifying circulating antigen levels during PCM can establish a correlation between antigenemia and the clinical evolution of the disease. This underscores the importance of integrating antigen detection in diagnostic and prognostic strategies for effective disease management.

Although the association between antigenemia and disease progression is well established in paracoccidioidomycosis, to the best of our knowledge this is the first study to investigate the potential relationship between antigenemia and oral lesions in *P. brasiliensis* hosts. However, the experimental conditions of this study found no oral lesions in any evaluated group, which prevented a direct assessment of correlation between antigenemia and mucosal involvement. The absence of oral lesions likely reflects a methodological limitation inherent to the murine model rather than an absence of biological association.

The failure to associate *P. brasiliensis* antigenemia with oral lesions in both mouse strains and fungal lineage may stem from differing immune responses (which impact lesion manifestation) and the duration of infection in this study. The pathogenic potential of *P. brasiliensis* is linked to its interaction with host immune cells. Ferrioti, et al.^34^ (2013) have suggested that the antigen levels alone fail to reflect tissue damage due to the differential macrophage response and the overall immune profile in A/Sn and B.10A mice. Tavares, et al.^35^(2012) have indicated that dendritic cells from resistant A/Sn mouse elicit a stronger Th1 response than those from susceptible B10.A ones. This suggests that varying antigen recognition and processing abilities in dendritic cells influence hosts’ immune response to the fungus. Also, Abreu, et al.^36^ (2020) have noted that the activation of mesenchymal stromal cells by *P. brasiliensis* via toll-like receptors increases inflammation, which may complicate PCM pathogenesis. Thus, despite the detection of *P. brasiliensis* antigens in A/Sn and B10.A mice, oral lesions may fail to develop due to mesenchymal stromal cells heightened inflammatory state. This could exacerbate or redirect the immune response away from oral lesion formation.

Considering these previous findings, the results highlight the complexity of host-pathogen interactions, making it difficult to associate *P. brasiliensis* antigenemia with oral lesions in the specific mouse models.

Another methodological aspect that deserves consideration is the inoculation route in this study. The intraperitoneal route was intentionally chosen because it provides a highly controlled and reproducible systemic infection, ensuring uniform dissemination of *P. brasiliensis* and consistent antigenemia between experimental groups. This approach enables reliable longitudinal monitoring of circulating fungal antigens and antibodies, the primary objective of this study. Although this route fails to reproduce the natural respiratory pathway of human infection, it enables standardized infection kinetics and avoids the variability commonly observed with intranasal inoculation. Nonetheless, it likely limits mucosal colonization and the development of oral lesions as these are more frequently associated with chronic infection models, using natural or respiratory routes of exposure. Therefore, the intraperitoneal route should be interpreted as an experimental strategy optimized for systemic and immunological analyses rather than for reproducing mucosal pathology.

The results regarding the production of anti-*P. brasiliensis* antibodies show that the highest total antibody levels are found at later times after infection with the virulent (Pb18) isolate. They also show that the isolate with slight virulence (Pb265) produces low antibody levels, reflecting low antigenic loads. Borges, et al.^37^ (2024) have discussed how extracellular vesicles from virulent isolates of *P. brasiliensis* induce a more robust immune response; in line with the observation that higher total antibody levels are produced at later stages of infection with virulent isolates such as Pb18. In contrast, the lower immunomodulatory capability and reduced expression of virulence factors in extracellular vesicles from the less virulent isolate (Pb265) correlate with lower antibody levels and suggest a reduced antigenic load. Other human studies support the correlation between differing antibody responses, and the virulence of the isolates that the hosts are exposed to.^38,39^

Although beyond the purpose of this study, some conclusions on analyses of the chemical composition of the *P. brasiliensis* antigen circulating in mouse sera can be derived from our data . Various virulence factors in *P. brasiliensis*, such as specific glycoproteins and metabolic adaptations, significantly influence the ability of an organism to elicit immune responses in hosts, contributing to the overall pathology of PCM.^40^

*P. brasiliensis* expresses a major glycoprotein known as gp43, which serves as a critical antigen in hosts’ immune responses. Studies show that antigenemia from gp43 correlates with clinical forms of PCM, highlighting how variations in *P. brasiliensis* virulence impact the immune response in patients.^32^ However, in this study, we observed no antigenemia in the A/Sn mice inoculated with Pb265 isolate, thus suggesting that the gp43 may have no correlation with cellular immunosuppressive responses. Therefore, the virulence of the infecting *P. brasiliensis* isolate, but not specifically the gp43 component, is fundamental for antigenemia. Only the highly virulent Pb18 elicits circulating antigens, whereas Pb18 and Pb265 express similar contents of gp43. These findings corroborate those of Lenhard-Vidal, et al.^32^, who reported no gp43 in the antigens from a *Paracoccidioides lutzii* strain that was isolated from a PCM patient.

In summary, the intricate relationship between fungal virulence and antigenemia in PCM elucidates the multifaceted interactions between *P. brasiliensis* and hosts’ immune systems. The virulence characteristics of the pathogen, compounded by the resultant immune responses, dictate the clinical manifestations and overall pathogenicity of the disease. The methodological limitations of this study underscore the challenges in translating findings from current animal models to human oral PCM and highlight the need for alternative experimental approaches to better investigate the mechanisms underlying oral disease manifestations.

## Conclusion

Our data show that circulating *P. brasiliensis* antigens stem from a very severe form of PCM, reflecting an infection caused by a highly virulent *P. brasiliensis* isolate in a genetically susceptible mouse strain. The high levels of circulating *P. brasiliensis* antigens probably impair the cellular immune response in the susceptible mouse strain.

Regardless of antigenemia levels, fungal strain, or host susceptibility, some methodological aspects of this study seem to influence the development of characteristic lesions in the oral cavity.
